# Impact of the COVID-19 Pandemic on Utilization and Cost for Care of Pediatric ALL

**DOI:** 10.21203/rs.3.rs-3706388/v1

**Published:** 2023-12-11

**Authors:** Alex Hoover, Dave Watson, Paige Reimche, Lynn Tanner, Laura Gilchrist, Mike Finch, Yoav Messinger, Lucie Turcotte

**Affiliations:** University of Minnesota Medical School; Children’s Minnesota; Children’s Minnesota; Children’s Minnesota; Children’s Minnesota; Children’s Minnesota; Children’s Minnesota; University of Minnesota Medical School

**Keywords:** Pediatric, Acute Lymphoblastic Leukemia (ALL), Care, Cost, Utilization, COVID-19, Pandemic

## Abstract

**Objective:**

Acute lymphoblastic leukemia (ALL) is the most common childhood malignancy and requires a unique pattern of healthcare utilization including an acute/emergent presentation and an intensive initial 8 months of therapy followed by two years of outpatient treatment. The COVID-19 pandemic caused massive global disruptions in healthcare use and delivery. This report aims to examine the effects of the COVID-19 pandemic on the presentation, diagnosis and continued management of childhood ALL in regard to utilization and cost of care.

**Results:**

Utilizing a commercial insurance claims database, 529 pediatric patients were identified who were diagnosed with ALL and completed their initial 8 months of treatment between January 2016 and December 2021. New diagnoses were evaluated by era and demographics. Utilization was measured by COVID-related era as number of inpatient and outpatient encounters, inpatient days, and cumulative cost. None of these cost or utilization factors changed significantly during or shortly after the pandemic. These findings reinforce that the necessary care for pediatric ALL is largely inflexible and was unwavering despite the massive shifts in the healthcare system caused by the COVID-19 pandemic. This provides a valuable benchmark as we further examine the factors that influence the pandemic’s impact on health equity and access to care, especially in vulnerable pediatric populations. This is the first investigation of the effect of the COVID-19 pandemic on utilization and cost of care in pediatric cancer.

## Introduction

Acute lymphoblastic leukemia (ALL) is the most common cancer of childhood and adolescence, with approximately 3000 new cases diagnosed each year in individuals under age 18 years of age in the United States (US).(1) The COVID-19 pandemic affected healthcare use and delivery across the United States and internationally, with multiple studies showing decreased healthcare utilization in both the emergency and ambulatory setting, particularly in the early months of the pandemic.(2–4) A reduction in visits due to patient fear of infection and reduced access to typical care due to public health regulations led to dramatic reductions in the use of preventive and elective care, including cancer-related care, during the first and second quarter of 2020.(5) Pediatric ALL is a disease that requires unique healthcare utilization including elements of acute/emergent presentation, an intensive initial 6–8 months of treatment and nearly two years of lower intensity outpatient treatment. We sought to examine the effects of the COVID-19 pandemic on the presentation, diagnosis and continued management of childhood ALL in regard to utilization and cost of healthcare and to examine whether changes in use and cost differ by patient characteristics.

## Methods

Using de-identified commercial insurance data from the OptumLabs^®^ Data Warehouse, a cohort of patients with ALL was identified, aged 1–30 years and diagnosed between January 2016 and March 2021 in the United States. ALL diagnosis was confirmed based on initial ICD-9 and ICD-10 diagnostic codes (204.00, 204.01, 204.02 and C91.00, C91.01, C91.02, respectively) in combination with bone marrow and lumbar puncture procedure codes within 14 days of diagnosis. Total number of new diagnoses were identified and stratified by timing of diagnosis, including early pre-COVID era (1/2016–6/2019), overlap COVID era (7/2019–3/2020), and COVID era (4/2020–3/2021). New diagnoses were also evaluated by age, sex and region of the US for differences in diagnostic patterns. Utilization was measured as number of inpatient and outpatient encounters, inpatient days, and cumulative cost (inflation adjusted) for the initial 8 months of therapy. Associations of demographics and utilization outcomes with era of diagnosis were assessed using chi-square test (or Fisher exact test when necessary) and Kruskal-Wallis test. To compare utilization during the two COVID eras to pre-COVID utilization, linear regression was used with robust standard errors.(6) Regression models for costs were performed on the log scale and Winsorized at the 1st and 99th percentiles to limit the impact of extreme outliers. R was used for statistical analyses.(7)

## Results

Among the 529 identified pediatric ALL patients diagnosed within the predetermined timeframe, 42.5% were female and median age at diagnosis was 7 years (interquartile range 4–15 years). There were 363 new ALL diagnoses in the pre-COVID era, 63 in the overlap COVID era, and 103 in the COVID era, and these frequencies were proportional to the length of the respective eras (p = 0.29). Patient characteristics of new ALL diagnoses across all three eras were similar with respect to age, sex, and geographic region (Table 1).

**Figure 1:**
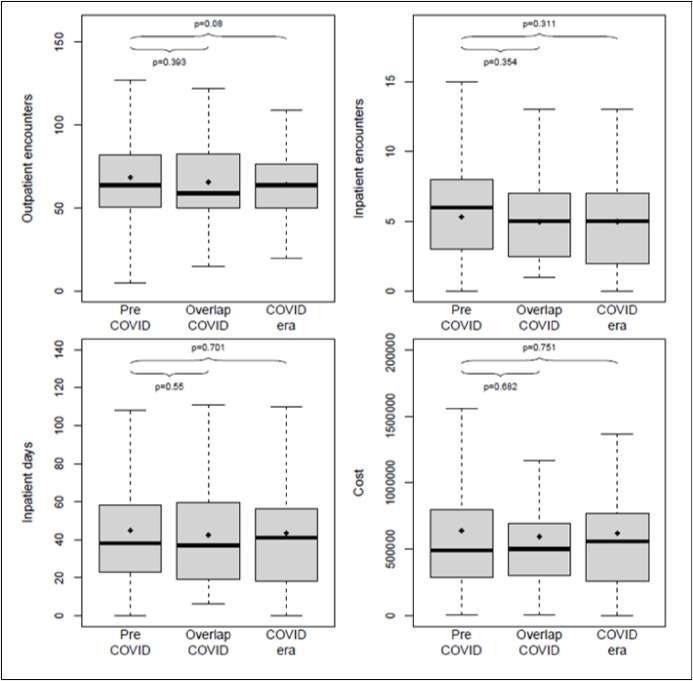
Inpatient/outpatient encounters, inpatient days and cost by COVID era (Pre-COVID era (1/2016 – 6/2019), overlap COVID era (7/2019 – 3/2020), and COVID era (4/2020 – 3/2021)).

Utilization for all four measures were similar across all three eras (Fig. 1). Over the first 8 months of therapy, the median utilization was 5 inpatient encounters, 64 outpatient encounters, 38 total inpatient days, and $495,000 for cumulative cost of care; these values did not vary significantly by era (all p > 0.05, Table 1).

## Discussion

This robust real-world cost analysis shows for the first time the effect of the COVID-19 pandemic on patterns of care for pediatric ALL – encompassing not only the pattern of new diagnoses but also patterns of care and cost during the initial 8 months of intensive therapy. We show that the number of inpatient and outpatient encounters, inpatient days and cost of care did not change significantly during the pandemic, reinforcing the fact that the necessary care for pediatric ALL is largely inflexible and was unwavering despite the massive shifts in the healthcare system caused by the COVID-19 pandemic.

Investigations into pandemic-related changes in care for other pediatric diseases that require significant medical care and attention at diagnosis include diabetic ketoacidosis (DKA) for new-onset type 1 diabetes have found an increase in incidence of DKA and severe DKA.(8) Shifts in access to primary care and patient fear of infection with accessing the healthcare system have been attributed to these alterations in diabetes presentation and DKA severity.(9)

However, in the face of major decreases in preventative and elective healthcare shown in other studies, we have shown that care for pediatric ALL continued at a steady rate throughout the early pandemic time period. This study provides a valuable benchmark of patterns that can be reassessed over time as we attempt to further examine the factors that influence the pandemic’s impact on health equity and access to care, especially in vulnerable pediatric populations.

### Limitations

Despite the many strengths of this study, limitations must be acknowledged. The use of claims data relies on accurate and consistent coding, which can make it difficult to ascertain the exact date of diagnosis or relapse. We were unable to comprehensively evaluate racial or ethnic differences in pandemic-era ALL cost or care utilization given the large proportion of individuals with missing or undefined race and ethnicity data. Additionally, the OptumLabs Data Warehouse is limited to commercially insured individuals, thus excluding publicly insured or managed care patients from this analysis and limiting the generalizability of these results.

## Figures and Tables

**Table 1:** Patient Characteristics and Utilization Measures by Era in Which ALL Diagnosis Occurred

Characteristic		Overall N = 529	PreCOVID N = 363	Overlap COVID N = 63	COVID era N = 103	p-value
Median age, years (Q1, Q3)		7 (4, 15)	7 (4, 16)	8 (4, 17)	7 (4, 14)	0.72
Sex, n (%)	Female	225 (42.5)	148 (40.8)	30 (47.6)	47 (45.6)	0.46
	Male	304 (57.5)	215 (59.2)	33 (52.4)	56 (54.4)	
Region, n (%)	Midwest	149 (28.2)	100 (27.5)	18 (28.6)	31 (30.1)	0.62
	Northeast*					
	South	219 (41.4)	148 (40.8)	29 (46)	42 (40.8)	
	Unknown*					
	West	98 (18.5)	74 (20.4)	7 (11.1)	17 (16.5)	
Median inpatient encounters (Q1, Q3)		5 (3, 7)	6 (3, 8)	5 (2.5, 7)	5 (2, 7)	0.48
Median inpatient days (Q1, Q3)		38 (21, 58)	38 (23, 58)	37 (19, 59.5)	41 (18, 56)	0.84
Median outpatient encounters (Q1, Q3)		64 (51, 81)	64 (51, 82)	59 (50, 82.5)	64 (50.5, 76.5)	0.62
Median costs, $1000 (Q1, Q3)		495 (282, 783)	491 (286, 797)	501 (298, 690)	560 (257, 770)	0.92

* Blank cells correspond to censoring cells; multiple cells need to be blanked so frequencies cannot be recovered, per OptumLabs^®^ guidelines

## Abbreviations

ALL: Acute lymphoblastic leukemia
US: United States
ICD: International statistical Classification of Diseases
DKA: Diabetic ketoacidosis
